# The New Italian Guidelines for Assisted Reproduction Technologies (ART): Ethical and Medico-Legal Issues

**DOI:** 10.3390/healthcare13020195

**Published:** 2025-01-19

**Authors:** Costanza Raimondi, Claudia Casella, Emanuele Capasso, Laura L. Campanozzi, Giulia Mercuri, Antonio Oliva, Antonio G. Spagnolo, Pietro Refolo

**Affiliations:** 1 Department of Health Care Surveillance and Bioethics, Section of Bioethics and Medical Humanities, Università Cattolica del Sacro Cuore, Largo F. Vito 1, 00168 Rome, Italy; costanza.raimondi1@unicatt.it (C.R.); antoniogioacchino.spagnolo@unicatt.it (A.G.S.); 2 Department of Advanced Biomedical Science, Legal Medicine Section, Università degli Studi di Napoli “Federico II”, Via Sergio Panini 5, 80131 Naples, Italy; claudia.casella@unina.it (C.C.); emanuele.capasso@unina.it (E.C.); 3 Research Unit of Bioethics and Humanities, Università Campus Bio-Medico di Roma, Via Álvaro del Portillo 21, 00128 Rome, Italy; l.campanozzi@unicampus.it; 4 Department of Health Care Surveillance and Bioethics, Section of Legal Medicine, Università Cattolica del Sacro Cuore, Largo F. Vito 1, 00168 Rome, Italy; giulia.mercuri01@icatt.it (G.M.); antonio.oliva@unicatt.it (A.O.); 5 Unit of Legal Medicine, Fondazione Policlinico Universitario A. Gemelli IRCCS, Largo F. Vito 1, 00168 Rome, Italy

**Keywords:** assisted reproduction technologies, guidelines, post-mortem embryo transfer, ex-partner embryo transfer

## Abstract

**Background:** On 20 March 2024, the Italian Minister of Health, in collaboration with the Italian National Institute of Health (Istituto Superiore di Sanità) and the National Health Council (Consiglio Superiore di Sanità), issued updated guidelines for assisted reproduction technologies (ART). They introduced two key changes: (1) permitting post-mortem embryo transfers, allowing a woman to proceed with the procedure after her male partner’s death, and (2) permitting embryo transfer even if the male partner is alive but the relationship has ended. **Objectives**: This study explores the ethical and medico-legal challenges posed by the updated Italian ART Guidelines, with a specific focus on the complexities of informed consent and the ethical dilemmas introduced by these provisions. **Methods**: This study employs a comprehensive review of legislation, case law, and a comparative analysis of national and international norms. Primary and secondary sources include laws, regulations, court decisions, and key ethical and medico-legal perspectives. Results were organized into two thematic areas: the medico-legal dimension, focusing on informed consent, and the ethical dimension, addressing post-mortem and ex-partner embryo transfers. **Results**: The new guidelines exacerbate the already intricate ART landscape in Italy. Post-mortem and post-relationship embryo transfers raise significant ethical concerns and amplify legal uncertainties. **Conclusions**: These updates highlight the pressing need for legislative reform to address unresolved ethical and legal issues in ART.

## 1. Introduction

In Italy, assisted reproduction technologies (ART) are regulated by Law 40/2004, known as the Norms on Medically Assisted Procreation [1]. This legislation has been instrumental in shaping the framework for ART in the country.

However, since its enactment, the law has faced criticism by some for being overly conservative [2,3] or excessively focused on safeguarding embryos [4], prompting extensive public debate and numerous legal challenges.

Public reaction to the law was immediate: in 2005, just a year after its enactment, five referendum questions were proposed to amend or repeal the law altogether. While the Constitutional Court deemed the proposal to abrogate the entire law unconstitutional (Ruling no. 45, 13–28 January 2005), it admitted the other four referenda. Thus, on 12–13 June 2005, Italian citizens voted on the following issues: restrictions on research on embryos; regulations on access restrictions to ART; norms addressing objectives; the rights of the subjects involved and access limitations; and the prohibition of heterologous fertilization (Rulings nos. 46, 47, 48, and 49 of 13–28 January 2005). However, voter turnout fell below 50%, making the referendum impetus fail and leaving the law in place: this was just the beginning of a prolonged battle [5].

Initially, access to ART was limited to heterosexual couples suffering from infertility or sterility, with both partners being over 18 years of age, alive, and either married or co-habiting (art. 1, Objective of the law). The law permitted fertilization of up to three embryos per cycle, with the requirement being to proceed with the transfer of all three of them simultaneously (art. 14, Limits to the application of techniques on embryos). This was because cryopreservation of embryos was restricted to cases where immediate transfer was not possible due to serious conditions or concerns for the woman’s health. Moreover, research on embryos was strictly prohibited (art. 13, Research on human embryos).

**Figure 1:**
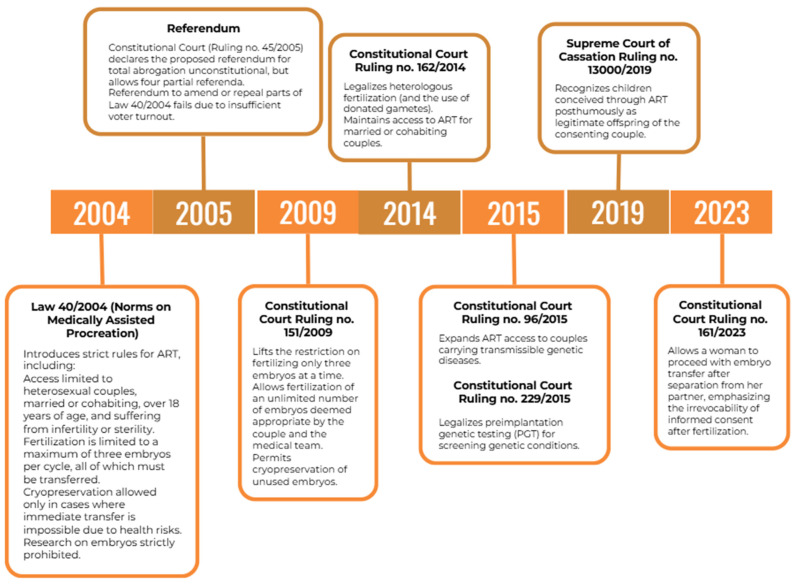
Timeline of legislative changes regarding ART in Italy.

Over the last twenty years, the law has undergone significant amendments, as shown in Figure 1. The most prominent include: allowing the fertilization of an unlimited number of embryos (as many as deemed adequate from the couple and the responsible doctor); consequently, allowing couples not to transfer embryos all at once and allowing cryopreservation for those embryos not immediately transferred (Constitutional Court Ruling no. 151, 2009); legalization of heterologous fertilization (thus introducing gamete donation), although the receivers must still be married or co-habiting couples (Constitutional Court Ruling no. 162, 2014). Couples who can access ART must be sterile, infertile or carrying a transmittable genetic disease (Constitutional Court Ruling no. 96, 2015), and preimplantation genetic testing (PGT) is allowed (Constitutional Court Ruling no. 229, 2015) [6]. These changes were made through court rulings and reflected in updated guidelines.

Indeed, art. 7 (Guidelines) of Law 40/2004 states that the Minister of Health, with the advice of the Italian National Institute of Health (Istituto Superiore di Sanità) and of the National Health Council (Consiglio Superiore di Sanità), should issue decrees containing new guidelines regarding ART adapting to scientific and technical developments. Given the fast speed of such developments, the law indicates that such guidelines should be issued at least every three years.

After a 9 year gap [7], on 20 March 2024 the Minister of Health issued the new guidelines, which were then published in the Official Gazette on 9 May 2024 [8].

This contribution aims to explore the ethical and medico-legal issues arising from these updates. Specifically, it will focus on the unique medico-legal challenges posed by the specific form of informed consent required by the procedure and will examine the ethical dilemmas stemming from two notable novelties: post-mortem embryo transfers and transfers following the dissolution of the couple’s relationship.

## 2. Materials and Methods

This study employs a comprehensive review of pertinent legislation, case law analyses, and a comparative examination of national and international norms. Both primary and secondary sources were used, including national laws, regulations, and guidelines in effect at the time of the study; relevant Constitutional Court and civil court decisions concerning ART; and examination of key ethical and medico-legal perspectives from professional associations, bioethics committees, and the academic literature.

The results were synthesized to evaluate the impact of the new ART Guidelines on existing medico-legal frameworks and ethical norms.

The findings were organized into two thematic areas: the medico-legal issues, with an emphasis on informed consent, and the ethical concerns, focusing on post-mortem and ex-partner embryo transfers.

## 3. Results

### Key Updates Introduced by the 2024 ART Guidelines

The 2024 Guidelines incorporate numerous changes, some of which had already been introduced through rulings by the Constitutional Court. Key provisions include the cryopreservation of genetic material for individuals seeking to preserve their fertility due to medical reasons (such as those affected by oncological, genetic, autoimmune, endocrine and surgical causes, as well as hematological pathologies); access to ART for couples who are not affected by infertility but are carrying transmissible genetic diseases; prohibition of experimentation on human embryos; permission to use PGT to screen for genetic conditions and to select embryos; and, notably, irrevocability of the informed consent once embryos are fertilized. In particular, the provision on informed consent raises significant ethical and medico-legal issues.

Art. 6 of Law 40/2004 concerns informed consent and specifies that “before recurring and in every phase of the application of the techniques of in vitro fertilization the doctor must inform in detail the subjects about the methods, the bioethical issues and the possible side effects and psychological effects that may come from such practice, on the chance probability and on the risks, as well as on the judicial consequences on the woman, the man and the to-be-born” [1]. In line with the rationale of the law, the article further mandates that couples be informed of alternative options for having a child, such as adoption or foster care, as regulated by Law 184/1983 [9].

Point 5 of the new guidelines, addressing informed consent, explicitly references the provisions of art. 6. To ensure that the couples’ will and consent is fully informed, the guideline emphasizes the requirement for joint written consent, provided by the couple to their doctor (or a representative). Furthermore, a mandatory waiting period of at least seven days must elapse between the moment consent is given and the initiation of the procedure (fertilization). This is crucial because, as reiterated in the 2024 Guidelines, consent becomes irrevocable once fertilization occurs, a principle that has been integral to the law since its inception.

The provision in Article 6 of Law 40/2004, which established that consent cannot be revoked after fertilization, was primarily intended to protect the embryo. This aligns with the law’s aim to prevent the destruction or cryopreservation of embryos (originally prohibited under art. 14, para. 1) that might result if one or both individuals changed their mind after fertilization. However, this provision raised significant concerns since its introduction and continues to do so. One major issue is that, while the law prohibits the withdrawal of consent, it does not establish penalties for non-compliance. Another significant concern arises from the implication that the impossibility for a woman to withdrawal of consent after fertilization would mean forcing the continuation of the procedure, including transferring the embryo to the woman’s uterus. This outcome is problematic and unfeasible for numerous reasons. Firstly, according to art. 32, para. 2, of the Italian Constitution, “no one can be forced to undergo a specific medical treatment except as provided by law” [10]. Secondly, imposing an embryo transfer would conflict with Law 194/1978 on voluntary termination of pregnancy [11], which safeguards a woman’s health and permits abortion when her physical or mental well-being is at risk, including situations stemming from conception. Forced embryo transfer and the resulting pregnancy would undoubtedly endanger a woman’s health, potentially leading her to seek an abortion immediately after. Thirdly, Law 219/2017 on informed consent and advance healthcare directives [12] specifies that no medical treatment can commence or continue without the free and informed consent of the individual, except when explicitly mandated by law. This implies that individuals can withhold or withdraw their consent for any healthcare treatment at any moment.

The only exception to the prohibition on withdrawing consent after fertilization pertains to cases involving PGT, where an embryo is diagnosed with a pathology that meets the severity criteria outlined by Law 194/1978 [11]. The rationale here is to allow a woman to decline transferring an embryo in her womb if she would proceed to abort it immediately after. This exception was not originally in the law, as PGT was only declared lawful in 2015, enabling this specific circumstance.

As said, initially Law 40/2004 did not allow for the withdrawal of informed consent after fertilization. However, the transfer would occur shortly after fertilization, with cryopreservation reserved for complex cases in which immediate transfer was medically unfeasible for the woman due to health concerns. Consequently, the temporal gap between fertilization and transfer was negligible, as was the time lapse between when consent was given and the embryo was transferred into the maternal womb. Now, it is up to the discretion of the doctor and of the couple to determine the appropriate number of embryos to fertilize and whether to cryopreserve them for a later transfer. This creates the potential for a significantly extended and non-negligible gap between fertilization and transfer.

For these reasons, in the final paragraph of the section on informed consent, the new guidelines legitimize two new scenarios: first, a woman can request to proceed with the transfer of the embryo after the male partner’s death (post-mortem), and, second, she can request the transfer also after the relationship with the male partner has ended.

In the post-mortem case, the guidelines align with the Supreme Court of Cassation’s 2019 decision (Ruling no. 13000, 15 May 2019) [13]. This case concerned a child conceived abroad through ART after the father’s death who was legally recognized as the offspring of the couple who donated their genetic gametes and provided informed consent for the procedure. According to art. 8 (Legal status of the newborn) of Law 40/2004, children born through ART must be legally considered the children of the couple who gave their consent under Article 6. Moreover, this legal recognition applies even if the couple does not meet certain conditions stipulated in the law, such as being married or co-habiting, being of different sexes, being over 18 years of age, and alive (art. 4).

The woman, after having conceived abroad, gave birth to the child in Italy and sought to register her child with the (deceased) father’s last name, which was initially denied by the public official. Without delving into the details of the legal proceedings, the case landed in front of the Supreme Court of Cassation, which ultimately ruled in favor of the woman, declaring the child to be the legitimate offspring of the couple who expressed their intent and consent. The child was allowed to bear her father’s last name with all the implications. The ruling underscores the “absolute central role of consent as a determining factor in parenthood, as it concerns those who are born out of ART” [13], in compliance with Article 8 of Law 40/2004.

The second scenario involves couples that are no longer married or co-habiting when the transfer request is made. In this case, clearly, it is the woman who seeks to proceed with the transfer without her ex-partner’s approval. The issue was addressed by the Constitutional Court regarding a case of a married couple who had fertilized embryos in 2017, following joint consent. However, no transfer occurred immediately after fertilization due to the woman’s health issues: she had to undergo some pharmacological treatments, more medical tests and checkups, as well as a so-called “endometrial scratch” to enhance the likelihood of success. In 2018, the husband left, and their separation was finalized in 2019. Nevertheless, in 2020 the woman requested to proceed with the transfer at the clinic where their embryos were stored. The man and the clinic found themselves in an unpredicted situation: both of them opposed her request. However, the situation was not fully unpredictable since Law 40/2004 already prohibit withdrawal of consent after fertilization.

The case reached the Constitutional Court, which, in Ruling no. 161, 2023, acknowledged the “tragic choice” involved in balancing the rights of the three subjects involved (i.e., woman, man, embryo). The court ultimately ruled in favor of allowing the woman to proceed with the transfer.

The court’s response indicated that the irrevocability of paternal consent is legally sustainable and does not violate the constitution. The court presented three key arguments to support the view that not allowing withdrawal of consent after fertilization is constitutionally valid [14].

(1) When the man expressed his consent, he was aware that the process could occur years later due to the possibility of cryopreservation. Consequently, he acknowledged the possibility of becoming a father long after initially giving his consent, even if the couple’s shared parenthood plan had changed in the interim. Additionally, his consent carries significant implications for other constitutionally relevant interests, particularly those of the woman. Indeed, as a result of their joint consent, the woman undergoes extensive and often invasive therapies and procedures over an extended period, which can pose substantial health risks. A woman’s involvement in the process of achieving pregnancy is inherently more intense than that of a man, as she carries the pregnancy with all its consequences on her body, and inevitably, herself as a whole. This disparity is exacerbated in the context of ART: a woman pursuing this path typically undergoes numerous medical evaluations due to difficulties in achieving pregnancy naturally. These medical evaluations and interventions continue throughout the process, and they may involve ovarian stimulation, hormone intakes, and egg retrieval, which is a procedure performed under general anesthesia. The psychological and emotional demands are also high and are heightened by the uncertainty of the result. While the emotional involvement is significant for the man too, his physical engagement is limited to sperm retrieval. Any potential withdrawal of consent would have particularly severe consequences if the woman, due to her age or health conditions, was no longer able to pursue another ART attempt, thereby potentially losing her autonomy over reproduction. Therefore, Law 40/2004 establishes the irrevocability of informed consent to safeguard the woman’s psychophysical integrity, as halting the process after fertilization could have detrimental effects on her mental and physical health.

(2) The irrevocability of paternal consent does not violate the principle of equality or constitute gender-based discrimination because the roles of women and men differ significantly. The man’s involvement concludes with the fertilization of the ovum, whereas the realization of the shared parenthood project requires the woman to undergo demanding cycles of ovarian stimulation and other interventions. In the ART process, the woman’s involvement far exceeds than the man, engaging both her body and psyche extensively. On this point, the court draws a comparison between assisted procreation and voluntary termination of pregnancy, as regulated by Law 194/1978. In both instances, men and women’s rights are different. A man’s will is subordinate, as a woman’s decision to terminate a pregnancy effectively precludes him from becoming a father. Similarly, a man who does not wish to have a child with a woman cannot impede her decision to continue a pregnancy. This distinction is justified by the fact that pregnancy exclusively involves the woman’s body. Neither continuation nor termination of pregnancy can be imposed upon her. Therefore, the man’s desire to halt the transfer of an embryo in utero holds no legal significance [14].

(3) ART are intended to “promote life” by fostering new births rather than merely achieving fertilization. The shared parenthood plan is realized through the fertilization of an embryo; the embryo is acknowledged by the court as being “more than mere genetic material”. This perspective is consistent with the principles of Law 40/2004, which has always sought to balance the rights of all parties involved, including embryos. When faced with the choice between not being born and being born into a family of separated parents (who remain parents), the latter is deemed preferable. The parents’ separation does not preclude the child from forming emotional bonds with both. Consequently, even in situations where the couple’s relationship has ended, prioritizing the woman’s request to implant the embryo, even after an extended period of cryopreservation, is considered necessary to protect the embryo’s primary interest in being born [14].

## 4. Discussion

### 4.1. Medico-Legal Issues of Informed Consent in ART

In 2017, the Italian Parliament enacted Law No. 219, which established a comprehensive framework for the practice of informed consent, aiming to protect patient autonomy and define the boundaries of medical consent [12]. This legislation set forth detailed provisions to ensure informed and voluntary decision-making in healthcare contexts.

Examining this law alongside principles developed through legal doctrine highlights the unique features and challenges of informed consent in ART. These challenges stem from the distinctive nature of ART, posing ethical, legal, and procedural complexities not typically encountered in standard medical practices [15]. Firstly, a core principle of informed consent is its timeliness, ensuring that the patient’s decisions at the time of a medical procedure reflect their current and informed will [16]. This principle safeguards individual autonomy, especially in cases where circumstances may evolve over time. In ART, however, consent acquires a distinct characteristic due to its delayed implications. Unlike standard medical interventions, where outcomes are immediate, ART often involves decisions—such as the storage or use of embryos—that materialize months or even years later. This temporal gap introduces significant questions, particularly when personal circumstances, such as relationship or health conditions, shift over time.

Secondly, informed consent for ART, as expressed by both partners, transcends a mere agreement to undergo a medical procedure. It constitutes a profound and binding declaration of the intent to become parents—a decision that becomes irrevocable once an embryo is fertilized [17]. Unlike standard informed consent for medical treatments, which can be generally withdrawn at any time, consent for ART serves as a cornerstone for the creation of human life and, from a strictly legal perspective, establishes parental rights and responsibilities. The irrevocability of this consent, once fertilization has occurred, establishes the legal status of the resulting embyo and child. This unique characteristic underscores the importance of ART consent, as it not only formalizes but also embodies the essence of parenthood and its associated responsibilities. Consequently, conflicts may arise when one partner seeks to withdraw consent after fertilization, but the law prioritizes the child’s established status and the original commitment over individual reconsideration.

Thirdly, consent for medical treatment must be informed: it requires the physician to divulge medical information, including aspects such as the patient’s health condition, available treatment options, potential benefits, and associated risks. This ensures that the patient’s decision is based on a clear and comprehensive understanding of their circumstances and the possible outcomes of different medical paths.

In the case of ART, however, the scope of information that the physician has to deliver extends far beyond medical details, necessitating a multidisciplinary approach that integrates legal, psychological, bioethical, and even existential aspects of the procedure [18]. Additionally, physicians are expected to ensure that the couple truly comprehends the complexity of the information provided. This requirement places a significant burden on healthcare professionals and raises a critical question: is the physician genuinely equipped to fulfill this expanded role? The challenge is further complicated by the risk of omission or inadequate information, which may result in legal claims. While Italian Law 40/2004 addresses some of these risks by ensuring that the parental status (status filiationis) remains intact even in cases of informational, it does not eliminate the risk of potential claims for inadequate or omitted information.

Lastly, informed consent is inherently personal. The legal act of consent is designed to protect the individuals and their right to therapeutic self-determination [18]. This safeguards against any form of public or private coercion, ensuring that medical decisions are made freely and autonomously. However, in ART, the right to self-determination is exercised jointly by the couple, irrespective of which partner is affected by sterility or infertility. This shared decision-making reflects the mutual involvement and responsibility required, adding complexity to balancing individual autonomy with collaborative commitments. For instance, if one partner’s medical condition or personal preferences significantly influence the couple’s choices, conflicts may arise, particularly when their priorities diverge.

In conclusion, informed consent in ART embodies unique medico-legal complexities that go far beyond those encountered in standard medical practice. The principles of timeliness, irrevocability, comprehensive information, and joint autonomy reveal the intricate interplay between patient rights, legal obligations, and the profound ethical considerations inherent in ART. These challenges emphasize the need for ongoing legal refinement and multidisciplinary collaboration to ensure that informed consent in ART effectively protects individuals while addressing its distinctive implications.

### 4.2. Ethical Issues of Post-Mortem and Ex-Partner Embryo Transfers

The 2024 Guidelines legitimized two scenarios: a woman may request to proceed with the transfer if the male partner is deceased (post-mortem) or if the male partner is still alive but their relationship has ended in the interim.

The first scenario raises numerous ethical questions, some of which have been addressed in the literature on post-mortem sperm retrieval [19]. However, the focus here is on a single issue, often referred to as “planned orphanhood” [20]. In ordinary pregnancies, conceived naturally without medical intervention, a child may tragically be born an orphan. In such cases, the pregnancy begins with both parents alive and the orphanhood results from unforeseen circumstances, such as natural disasters, wars, acts of terror, illness, accidents, or other unpredictable events. While deeply unfortunate, these outcomes occur independently of any deliberate human intervention.

In contrast, ART challenges traditional ethical principles of reproduction by transforming the role of external professionals from merely enabling life to actively shaping its conditions. Within this framework, orphanhood is no longer the result of unforeseen tragedy but instead arises directly from deliberate reproductive decisions [20]. This raises fundamental ethical dilemmas, such as does an adult’s desire to bring an orphaned child into the world take precedence over the child’s basic right to have two living parents at birth? Can such an aspiration justify creating a person who will never have the opportunity to know the absent parent? Is the desire for parenthood fueled by grief over the partner’s loss, or is it genuinely independent from that loss? Framed differently, the question could become one of two forms of suffering: the pain of an adult who has lost her partner and the opportunity for a shared descendant versus the pain of a child whose orphanhood is predetermined at birth.

The behavior and emotions of individuals who have recently experienced the loss of a loved one are well documented in the literature [21,22]. When a partner dies, the surviving one often needs time to emotionally detach from the deceased—a painful process marked by loneliness that other social relationships cannot alleviate. Within this context, the desire for parenthood after the partner’s death presents complex challenges. Individuals seeking to have a child after such a loss may wrestle with two conflicting desires: on the one hand, the desire for continuity and the wish to maintain life as though the loss had not occurred; on the other, the yearning to memorialize the deceased, using the creation of a new life as a way to honor and preserve their memory.

In line with the well-known Kantian principles advocating respect for intrinsic human dignity, these circumstances may pose a risk for the unborn child in terms of being regarded not as an end in itself but rather as a means to fulfill the purposes or desires of others. This imposes a moral obligation on those who choose to conceive, requiring them to ensure that the child is provided with the necessary conditions for a harmonious upbringing. Failure to do so places the child in a vulnerable position. The phenomenon of “planned orphans”, referring to children intentionally born following the death of one parent, remains underexplored in contemporary research. Comprehensive studies assessing how these children navigate the psychological complexities of growing up in the shadow of parental loss, particularly when this loss precedes their birth by some time, are lacking. This knowledge gap leaves significant questions unanswered about the long-term emotional and behavioral outcomes of this unique group.

Some studies, however, suggest that children in single-parent households may face an elevated risk of emotional and behavioral challenges, including anxiety, depression, and difficulties in forming social relationships [23,24,25]. Nevertheless, this remains a subject of ongoing debate and further investigation [26]. Therefore, addressing the question posed here is challenging and leaves many issues unresolved.

Many of the considerations outlined above can be extended to the second scenario as well. In this context, ART once again transforms the role of external professionals from passive enablers of life to active participants in shaping the child’s existence. This shift highlights the profound ethical and social implications inherent in such interventions.

Even though this topic is controversial, parental rejection, characterized by a lack of warmth, acceptance, or validation, creates an environment in which children may internalize negative self-perceptions. This internalization often manifests as shame—a deep sense of inadequacy and unworthiness. Studies suggest that this dynamic may impair emotional development, social functioning, and long-term mental health [27,28]. As parental rejection remains a significant determinant of a child’s psychological health, understanding its roots and consequences becomes essential—not only to address the immediate needs of children but also to evaluate the ethical dimensions of reproductive choices. Therefore, a critical ethical question emerges again: does the mother’s desire to procreate precede a history of “planned vulnerability” for the child?

These concerns align with the critique of German philosopher Jürgen Habermas, who argues that a “designed” individual might not feel entirely free or equal to others. Such an individual could perceive their existence as the product of “parental decisions” rather than the result of chance, as is the case with natural conception. This perception risks creating an ethical disparity between those conceived naturally and those who were “planned”, with profound implications for social relationships and personal autonomy [29,30]. Both scenarios discussed, involving the planning for parental absence or refusal, may amplify these concerns by introducing complex ethical challenges rooted in inequality, underscoring the need to address such disparities in a thoughtful and balanced manner.

## 5. Conclusions

Our analysis highlights how the 2024 Guidelines for ART introduce additional complexity to an already intricate subject. We acknowledge that our discussion is neither exhaustive nor definitive; it represents a small contribution—a “drop in the ocean”—to the broader discourse surrounding ART. This field is marked by profound ethical, legal, and social challenges stemming from the often-irreconcilable conflicts of interest it entails: the protection of a woman’s physical and psychological health, her autonomy to become a mother, a man’s right to autonomy, and the dignity of the embryo.

Frequent judicial rulings and modifications to Law 40/2004 make it evident that legislative intervention is needed. While courts in Italy can ensure adherence to laws or assess their constitutionality, the responsibility for enacting and reforming legislation lies with the parliament.

Therefore, a comprehensive legislative effort is necessary to address these multifaceted ethical, legal, and social dimensions, providing the clarity and balance required to effectively navigate this evolving and complex domain.

## Data Availability

Data are contained within the article.
